# Why Health Care Professionals Belong to an Intensive Care Virtual Community: Qualitative Study

**DOI:** 10.2196/14068

**Published:** 2019-11-05

**Authors:** Kaye Denise Rolls, Margaret Mary Hansen, Debra Jackson, Doug Elliott

**Affiliations:** 1 Centre for Applied Nursing Research University of Western Sydney Liverpool Australia; 2 Ingham Institute for Medical Research Liverpool Australia; 3 South Western Sydney Local Health District Liverpool Australia; 4 University of Technology Sydney Sydney Australia; 5 University of San Francisco San Francisco, CA United States; 6 Oxford Health NHS Foundation Trust Oxford United Kingdom; 7 Ngangk Yira Research Centre for Aboriginal Health & Social Equity Murdoch University Perth Australia

**Keywords:** social media, focus groups, physician, nurse, intensive care, innovation diffusion, scholarly communication

## Abstract

**Background:**

Clinical practice variation that results in poor patient outcomes remains a pressing problem for health care organizations. Some evidence suggests that a key factor may be ineffective internal and professional networks that limit knowledge exchange among health care professionals. Virtual communities have the potential to overcome professional and organizational barriers and facilitate knowledge flow.

**Objective:**

This study aimed to explore why health care professionals belong to an exemplar virtual community, ICUConnect. The specific research objectives were to (1) understand why members join a virtual community and remain a member, (2) identify what purpose the virtual community serves in their professional lives, (3) identify how a member uses the virtual community, and (4) identify how members used the knowledge or resources shared on the virtual community.

**Methods:**

A qualitative design, underpinned by pragmatism, was used to collect data from 3 asynchronous online focus groups and 4 key informant interviews, with participants allocated to a group based on their posting behaviors during the previous two years—between September 1, 2012, and August 31, 2014: (1) frequent (>5 times), (2) low (≤5 times), and (3) nonposters. A novel approach to focus group moderation, based on the principles of traditional focus groups, and e-moderating was developed. Thematic analysis was undertaken, applying the Diffusion of Innovation theory as the theoretical lens. NCapture (QRS International) was used to extract data from the focus groups, and NVivo was used to manage all data. A research diary and audit trail were maintained.

**Results:**

There were 27 participants: 7 frequent posters, 13 low posters, and 7 nonposters. All participants displayed an external orientation, with the majority using other social media; however, listservs were perceived to be superior in terms of professional compatibility and complexity. The main theme was as follows: “Intensive care professionals are members of ICUConnect because by being a member of a broader community they have access to credible best-practice knowledge.” The virtual community facilitated access to all professionals caring for the critically ill and was characterized by a positive and collegial online culture. The knowledge found was credible because it was extensive and because the virtual community was moderated and sponsored by a government agency. This enabled members to benchmark and improve their unit practices and keep up to date.

**Conclusions:**

This group of health care professionals made a strategic decision to be members of ICUConnect, as they understood that to provide up-to-date clinical practices, they needed to network with colleagues in other facilities. This demonstrated that a closed specialty-specific virtual community can create a broad heterogeneous professional network, overcoming current ineffective networks that may adversely impact knowledge exchange and creation in local practice settings. To address clinical practice variation, health care organizations can leverage low-cost social media technologies to improve interprofessional and interorganizational networks.

## Introduction

### Background

Modern health care is delivered in complex organizations by a range of health care professions. Significant clinical practice variations may exist [1,2] in part because of ineffective internal and professional networks that limit knowledge exchange between health care professionals (HCPs) [3,4]. Virtual communities (VCs) have the potential to overcome these professional and organizational barriers [5,6], facilitating knowledge flow between HCPs and across organizations. This was the final study in a multiple-methods research program, where 3 concurrent studies examined interrelated aspects of an exemplar VC (ICUConnect): (1) the professional social network [7] (2) community participation, including knowledge exchange (manuscript under review), and (3) why HCPs join and remain members (protocol; [8]). The aim of this study was to explore *why* HCPs belong to ICUConnect.

### Diffusion of Innovation

Everett Rogers [9] developed the *Diffusion of Innovation* (DOI) theory by integrating study findings from agriculture where researchers examined how individuals adopted innovations over time. Rogers then evolved the theory by undertaking studies across different countries and levels of economic and social development [9]. In health, these innovations could include new equipment, research findings, or practices. Early research focused on how the interplay between the relative characteristics of the innovation, time, and communication channels and structure of a social group affected the diffusion and adoption of that innovation over time. An innovation is an idea, practice, or object that is perceived to be new by an individual or work group, and there are 5 characteristics that influence this perception: relative advantage, complexity, compatibility, trialability, and observability [9]. Rogers found that for an innovation to diffuse across a social group, at least the first 16%, comprising innovators and the early adopters (visionaries), needed to adopt before a critical mass was reached and adoption spread to the early majority (pragmatists). The latter groups of late majority and laggards became interested in adoption when it was apparent; they were straying from group norms. A critical difference between early and late adopters is the former have greater access to new information because of the number and quality of communication channels they choose to maintain, especially outside their close social circles. For technology adoption, the gap (Moore’s chasm) between the visionaries (early adopters) and pragmatists (early majority) may only be crossed when proof of the technology efficacy has been demonstrated and championed by early adopters (see Multimedia Appendix 1) [10-11].

Contemporary research has demonstrated how organizational or group factors exert a powerful influence on both individuals and the organization [12-14]. There are 7 key internal organizational factors that influence an organization’s ability to develop or implement innovations, including centralization, complexity, formalization, interconnectedness, organizational slack, external orientation [9], and absorptive capacity [12,15]. Interconnectedness (connections between organizational members and units) and external orientation (organizational leaders with external networks) are both mediated by communication channels or networking internally or external to the organization [9,12-13]. Furthermore, an external orientation reflects an individual’s attitude toward change, which is an independent variable when evaluating the innovativeness of an organization [9]. Individuals with communication channels outside their everyday social and professional networks will have greater access to new information because they are crossing boundaries between social groups; however, unless the source is considered credible, the veracity of information will be questioned [16]. These boundary spanning activities are vital if an organization is to have access to novel information and innovations [17]. For further description of DOI, refer to Multimedia Appendix 2.

### Social Networks and Optimal Patient Outcomes

For patients to experience optimal outcomes, health care organizations must deliver clinical practices based on contemporaneous evidence. Effective identification and integration of knowledge requires organizations to balance a dense homogenous internal social network with low density diverse external social networks [12,16,18]. The prevailing vertical hierarchical structures, however, do not support the development of a cohesive, cooperative, and multidisciplinary culture necessary to address contemporary health care challenges [19]. The current reality is that significant clinical practice variability exists, leading to suboptimal patient outcomes [1]. This variability may be because of ineffective social networks [20-23] that restrict the flow of knowledge into and around a health care organization and onto individual HCPs. Some contributing factors have been identified including (1) the hierarchical organizational structure that isolates clinicians and restricts knowledge flow [4]; (2) professional boundaries between members of multidisciplinary teams that limit a shared understanding of specialty knowledge [24]; (3) workplace socialization forcing clinicians to comply with currently accepted practices [25,26]; and (4) HCPs from a range of disciplines who prefer knowledge sources that are human, easily accessible, and perceived to be credible [27-32]. There is increasing interest in the use of social media to create these social networks as VCs that have the potential to overcome the above barriers [5], so that HCPs have sustained access to novel knowledge [33,34].

### How Health Care Professionals Use Social Media to Form Virtual Communities

HCPs began using VCs in the early 1990s, although uptake of Web-based communication varies considerably across disciplines and specialties [7,35-37], and despite positive public attitudes toward what is today coined *social media*, this has not translated into significant professional use. At present, regardless of platform, the vast majority of VC members tend to not post or post infrequently; however, this is reversed when examining how often members access a VC or read posts [38]. At an individual level, members who post in an HCP VC are seeking a better understanding of the current knowledge and best practice in their particular field [39-41] or to assist fellow clinicians [36,40,42,43]. This suggests HCPs use VCs to establish virtual professional networks [13] to enhance access to colleagues and best practice knowledge. These members also develop a commitment to the VC and are motivated to post by collectivism [36,40,42,43], reciprocity [36,42,43], and where the Web-based environment is perceived to be respectful [42,43] and noncompetitive [44]. Members tend not to post when they lack time or interest, knowledge self-efficacy, confidence [41,42,45], or skills to use the platform [41,42] and when the Web-based culture or discussions are viewed unfavorably [36,41,45]. There are some data suggesting that this is influenced by individual characteristics [46], peers [46,47], and perceptions of the platform as an innovation [46]. Similar to nonhealth VCs, there is a symbiotic relationship between the online culture of a VC, members, and knowledge-sharing activities [48,49].

At present, the research base concerning the efficacy of health care VCs remains inadequate, as most of the studies concerning HCP VCs or on why or how HCPs use social media rely on Web-based observation [38], which only reveals the perspective of posters, who represent a minority of VC members. Given that regardless of professional group or industry, most VC members prefer to read rather than post [50,51], what is it that motivates HCPs to join a VC and what do they find of value that influences them to remain members? Ideally a member survey would provide data more representative of a whole community; however, prior research has struggled to obtain representative samples [52-57]. Therefore, a qualitative design was chosen because it would collect rich data from all types of members, especially the unrepresented nonposting majority. Understanding these phenomena will assist health care leaders in understanding how to develop and implement VCs to optimally leverage social media to improve knowledge diffusion and patient care.

### Study Aim

The aim of this study was to explore *why* HCPs belong to ICUConnect. The related research objectives were to (1) understand why members join and remain a member, (2) identify what purpose the VC serves in in their professional lives, (3) identify how a member uses the VC, and (4) identify how members used the knowledge or resources shared on the VC.

## Methods

### Design

A qualitative design underpinned by pragmatism [58,59] was developed with data collected using three asynchronous and nonanonymous online focus groups and key informant interviews with participants allocated to a group based on their posting behaviors in the previous two years. The different modes of community participation by members and the symbiotic nature of the relationship between members and an individual VC [38] suggest that there is no universal VC experience. At the core of pragmatism is the acceptance of pluralism [60,61], and the value of knowledge is intrinsically dependent on the social context and values of both the research participant and scientist [61]. A range of theories have been used to develop an understanding of how or why HCPs use VCs, including the theory of planned behavior [47,62], technology acceptance model [46], and community of practice (CoP) [39,43]. The DOI theory [9] was chosen as the theoretical lens because of the need to explore the intersection between the individual member, the organization, and the innovation (ICUConnect) rather than to identify the relative importance of individual aspects. The protocol for this study has been published [8].

### Ethics

A total of 2 approvals were obtained from the Human Research Ethics Committee (HREC) of the University of Technology Sydney. The first approval (HREC 2014000378) covered the online focus groups. For the online focus groups, participant confidentially was ensured by (1) a group rule, covering nondisclosure of participant names or sharing the content of posts, and participants agreed to abide when they registered for the study and (2) focus groups were convened within a secure website using a closed, password**-**protected discussion forum with the social media sharing function disabled. These layers were designed to ensure participant confidentiality and prevent forum posts from being searchable via the Web [63]. Informed consent for participants was included as part of the Web-based registration form. An amendment to undertake key informant interviews (HREC 2014000683) was granted because of a shortfall in recruitment for the frequent poster focus group. Participant identifying information could not be removed from the online focus groups’ text; however, it was removed from transcribed interviews. All participants were given a unique identifier number to maintain a link with their original data. Confidentiality of participants was maintained by storing original data including focus group data and interviews (as MP3 files) within a university-authorized secure cloud server (Oxygen). Participant deidentification was maintained using a standardized taxonomy.

### Setting

ICUConnect is a listserv, established in 2003 by a New South Wales Health state–based unit (the Intensive Care Coordination and Monitoring Unit) to provide intensive care (IC) clinicians with online network to exchange information and improve patient care [45]. At the time of data collection, there were approximately 1600 members from all health care professions who worked at about 225 health care facilities, universities, and industry partners. Although these HCPs were from several countries, the majority were from Australia with nurses being the largest professional group [7].

### Participants and Sample

A purposive stratified sampling method [64] was used to recruit the participants for the online focus groups and subsequent key informant interviews. The aim was to recruit 8 to 12 participants for each of the 3 focus groups [65,66], with focus group assignment based on Web-based participation over the preceding 2-year period (frequent: posting >5 times, low: posting ≤5 times, and nonposters: no posts). The rationale for this was to create a Web-based space where participants felt comfortable and confident that their contributions will be met in a positive and supportive environment because the other participants shared their preferred mode of participation; that is, a low or nonposter would not feel intimidated because there were no high posters who might monopolize the conversation [67]. An invitation to participate was posted on ICUConnect, with a link to the Web-based recruitment form (Google forms; Google). The recruitment form included participant information, consent, participant demographics, and a short survey covering group rules (Netiquette; refer to Multimedia Appendix 3). Once a potential participant had completed the registration and consent, their posting behavior was reviewed, and they were assigned to a focus group and notified. This review was completed by searching KDR’s email archive using the potential participant’s email address. Once located, the posting activities of the potential participant between September 1, 2012, and August 31, 2014, were evaluated.

### Data Generation

There were 4 sources of data: (1) 3 online focus groups, (2) key informant interviews, (3) research diary, and (4) the audit trail. The first 2 components are discussed in the following section, whereas the latter 2 are discussed in the Study Methods: Strengths and Limitations section.

### Moderating Focus Groups

Each focus group was conducted over 3 weeks between October and December 2014, using a closed discussion forum (IPBoard version 3 Invision, Powerboard) that was hosted on a secure jurisdictional health department website. The platform was chosen because it was accessible and usable across fixed and mobile devices. For each focus group, there were 2 weeks of active discussions, with each forum kept open for another week for any further comments. The focus groups were held in the following order: (1) low posters, (2) nonposters, and (3) frequent posters, with the low- and nonposting groups overlapping by a week.

The approach to focus group moderation was based on principles from moderating traditional focus groups [65] and facilitation of learning on the Web or electronic moderating [68] (see first table of Multimedia Appendix 4 for a priori moderating plan). KDR moderated the focus groups, and DE was a nonparticipant observer. This approach was developed a priori to maximize conditions for the development of rich data by facilitating optimal participation and interaction, and safeguarding participant confidentiality [65].

The focus group question guide was informed by *DOI* [9] and refined over time to reflect how discussions evolved (see second table of Multimedia Appendix 4 for question guide). Each question posted by the moderator formed a discrete discussion thread that explored a specific aspect of the VC including positive and negative aspects. A schedule was used with new questions posted every 2 to 3 days depending on activity. Participants were alerted to a new question by emails using a standard script with an informal and conversational tone. Elements of this standard script included the following: (1) expression of appreciation for participation, (2) reiteration that help was available if technical issues were experienced, (3) the question and any clarifying information, and (4) where applicable, summaries of previous posts that were germane to a new question.

### Key Informant Interviews

A total of 4 frequent posters were purposively recruited and interviewed, between February to June 2015, to address the shortfall in the number of participants in the frequent poster focus group. A total of 3 interviews were face-to-face as participants were in metropolitan Sydney, and 1 was conducted via Skype (Skype Communications SARL, Microsoft Corporation) as the participant was located outside this area. 

### Data Collection

Data collected included (1) demographic data describing participant characteristics, (2) categorical data describing discussion forum participation, (3) discussion threads documenting focus group discussion, (4) transcripts of key informant interviews, and (5) field notes and research diary. Field notes recorded what the researcher experienced during data collection and included (1) both a description of and reflection on what occurred, (2) reflections on personal thoughts and feelings, and (3) any insights, judgments, and interpretations made in the field [69]. Once collected, data were stored in an NVivo file (versions 10 and 11, QRS International).

Data from the 3 online focus groups were collected using NCapture (QRS International) and imported into NVivo. The 3 face-to-face interviews were recorded on a mobile phone whereas the Skype interview was recorded using an MP3 Skype recorder (Alexander Nikiforov). These MP3 files were transcribed via a Web-based service (Transcribe Me!); following this the transcripts were anonymized and imported into NVivo for analysis. Field notes were developed concurrently with the online focus groups and during data analysis using the memo function of NVivo. An interview sheet was used to make notes during the interviews, and this was scanned and imported into NVivo.

### Data Analysis

In keeping with the pragmatic realist approach, an analysis of focus group and key informant interviews was completed using Braun and Clarke’s 6-step thematic approach (this is expanded upon in Methods in Multimedia Appendix 4) [70]. DOI [9] was selected as the theoretical lens, as it aligned with both the broad problem of inadequate social networks limiting knowledge diffusion in health care, and current gaps in the literature. Member checking of early themes was undertaken during focus groups where responses could be seen to be converging.

### Researcher Bias and Relationship With Participants

KDR was the long-term moderator of the VC, and DE was a member; however, the other authors were not members or associated with the VC. To manage any potential for bias during data collection and analyses and to establish a welcoming nonhierarchical atmosphere, 2 key procedures were completed. KDR withdrew from the moderator role several months before participant recruitment and completed a bracketing process [71]. This formed a part of the research diary, and these assumptions were revisited during data analyses. To mitigate for possible coercion during the focus groups, nonauthoritative language was used, and the roles of researcher and moderator (KDR) and nonparticipant observer (DE) were made explicit.

## Results

This section reports study findings within the context of the DOI. The participants, including the participants as innovators, are described first, followed by ICUConnect as social media, and then presentation of the overarching theme of why HCPs belong to the VC. Participant contributions are reported verbatim except for correction of spelling and participant deidentification.

### Participants

A total of 29 members enrolled for the focus groups; however, only 23 participated. Overall, there were 27 participants for this study (7 frequent posters, 13 low posters, and 7 nonposters). For the frequent poster group there were 3 from the focus groups and 4 key informant interviews (see first table of Multimedia Appendix 5). All participants had significant experience as HCPs and IC clinicians, with frequent poster participants the most experienced (see second table of Multimedia Appendix 5). Their length of professional experience suggests that all participants were digital immigrants, that is, born before 1980 [72]. 

Participants from the posting groups exhibited stronger external orientation or boundary spanning than nonposters, as evidenced by the frequency with which they described sharing ICUConnect discussions with colleagues inside and outside their local working environment. Low and nonposters shared a lack of knowledge self-efficacy, a preference for offline communication, and being an observer. Knowledge self-efficacy or lack of (a feeling of not having the experience or knowledge to add to a discussion) was demonstrated by the following quote:

I am an observer for a number of reasons...I have worked for a number of years away from the floor of the ICU...feel that I am not right up to date with the latest clinical information in the area. In my general workplace demeanor, I am reserved but definitely not a passive person.

NUM FG2-6

Overall, 60% (16/27) indicated they used other social media. A total of 70% (5/7) of frequent posters reported professional use of other social media compared with just over 50% for low (7/13) and nonposters (4/7). Specialty-specific VCs (discussion forums or listservs) were the most common extra social media used (26%, 7/27), followed by ResearchGate (22%, 6/27), Twitter (19%, 5/27), and podcasts or YouTube (15%, 4/27). Facebook was commonly used for personal networking only (48%, 13/27).

### ICUConnect as Social Media

ICUConnect, an email-based listserv, was perceived by participants to be superior to other social media in terms of compatibility, complexity, and relative advantage (see Multimedia Appendix 2). Importantly, other social media were perceived as incompatible with professional values and beliefs because of the volume of information, the intrusiveness of nonprofessional information, or unprofessional language (eg, abbreviations). ICUConnect was also viewed as superior to (relative advantage) over other media because it was specific to the Australian IC context, queries were answered quickly, and the platform was perceived as being less complex to use, especially for technologically naive members.

### Theme—Why We Belong

**Figure 1:**
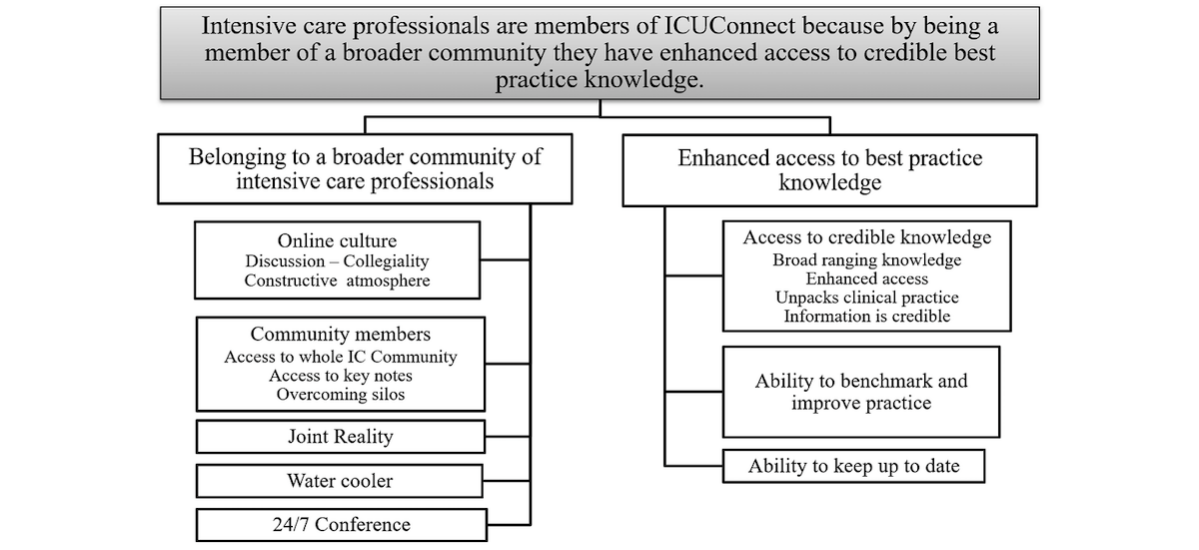
Main theme. IC: intensive care.

The overarching theme identified was that participants were members of ICUConnect because by belonging to a broader community of IC professionals they had enhanced access to credible best practice knowledge. A total of 2 subthemes were identified, each with elements that provided structure and context for the theme within the lens of DOI (see Figure 1): (1) *Belonging to a broader community of IC professionals* (short name: Belonging to a community) embodied the social system of ICUConnect and (2) *Enhancing access to best practice knowledge* (short name: Access to knowledge) represented how the VC facilitated innovation access for members.

#### Subtheme—Belonging to a Broader Intensive Care Community

This complex subtheme displayed 5 elements, as noted in Figure 1, and are discussed in further detail below. The online culture of ICUConnect was the largest subtheme element and highly valued by members regardless of posting behavior. This culture was characterized by informative discussions, collegiality, and a constructive atmosphere. Discussions were the dominant characteristic described and were viewed as being both highly and least valued by participants. When described positively, discussions were portrayed as informative and entertaining cross-disciplinary debates that provided valuable perspectives that were not available where participants worked (see Table 1, Exemplar 1). Conversely, participants also felt that discussions were limited by a lack of robust argument, nonevidence-based or ill-informed answers, that some members used discussions for self-aggrandizement, and that on occasion the content or intent of posts were misconstrued because of a lack of personal knowledge of a poster (see Table 1, Exemplar 2). The collegiality of ICUConnect was valued and was cited as a reason to join. This collegiality was exemplified by altruism (expressed by frequent posters), the willingness of members to share with colleagues, and that help was available when asked for. Importantly, this collegiality extended beyond nursing and medicine to include allied health members (see Table 1, Exemplar 3). Overall, participants felt that ICUConnect had a constructive, respectful, and informal atmosphere or tone that expedited access to knowledge, and importantly, lacked malicious interactions such as flaming or disparaging comments (see Table 1, Exemplar 4). Several participants, however, remained concerned regarding the reception of their posts (refer to third table in Multimedia Appendix 5 for more exemplars).

The second element of *Belonging to a community* was *community members*, which was characterized by 3 elements. Participants said that because ICUConnect provided *access to the whole of the IC community*, the VC made members feel a part of a broader community that simplified their networking (see Table 2, Exemplar 5). This facilitated access to IC experts (*keynotes*), which was highly valued and cited as a reason to read a post (see Table 2, Exemplar 6) and supported members in overcoming any clinical or practice silos created by local organizational structures (see Table 2, Exemplar 7). Refer to fourth table in Multimedia Appendix 5 for more exemplars.

The third element of belonging to a community was *joint reality*, where participants expressed feelings of being connected to the community, particularly when colleagues disclosed that they were experiencing similar clinical practice issues. For frequent posters this also created a sense of contributing to improving patient care on a broader scale (see Table 3, Exemplar 8). This element symbolizes a perceived homophily, that is, a sense of belonging to a like-minded group with shared values and experiences [9].

The fourth element was that ICUConnect functioned like discussions around a *watercooler* or an informal meeting place [73], where participants described using discussions to initiate conversations with work colleagues and reflect on local practices. This element was described most often by low posters but only occasionally by frequent or nonposters. As a watercooler space, ICUConnect was perceived as an extension of their local unit, with information that could be used locally or sparking and informing local discussions with new perspectives, ideas, and contemporaneous practice trends (see Table 3, Exemplar 9).

The final element was *24/7 conference*, which was a descriptor for ICUConnect because it provided immediate access to colleagues, research, and evidence; a circumstance normally limited to structured professional events such as annual conferences or seminars (see Table 3, Exemplar 10). The VC was, therefore, seen as superior or having a relative advantage over traditional professional events as it was always available and required no money or time to attend (see fifth table in Multimedia Appendix 5 for more exemplars).

**Table 1:** Belonging to a broader intensive care community: Element 1—online culture exemplars.

Online culture element	Exemplar
Exemplar 1: Positive discussion	*I think it’s the opportunity to speak to other colleagues, be that medical or nursing, and to drill down to some of the points...at the time, you know, we all had this good debate, and I think it—I think as the debate progressed, more people came in on that discussion, and I think the wider community hopefully benefited from that. So, I think having a dialogue is of benefit.* [Equipment manager KI-2]
Exemplar 2: Negative discussion	*In terms of negatives, all I can think of (and I really had to think!) is that some posts can be misunderstood if you do not know the person posting (especially for those who are new to ICUConnect). One might say that some would be discouraged from posting, fearing a “not so favorable” reply that is FOREVER there for the whole.* [Health care manager FG3-4]
Exemplar 3: Collegiality	*Since my role has changed, I have used ICUConnect a little more to seek out advice and ideas from other areas. Much of the responses have been very positive and I have enjoyed the sharing and caring.* [Clinical nurse external FG2-5]
Exemplar 4: Constructive atmosphere	*...and I think I like principally the respectful way that people—or that they visibly deal with queries and questions and so on. And I’ve seen a few kind of attempts to correct direction through the years, and they’ve all seemed to be received well and I've agreed with them all. So I guess that it’s a respectful environment that people feel really free to ask questions, sometimes over and over and over again.* [Knowledge broker KI-3]

**Table 2:** Belonging to a broader intensive care community: Element 2—community members.

Community members element	Exemplar
Exemplar 5: Whole of intensive care community	*ICUConnect provides me exposure to the ICU community; their thoughts; interests; discussions and topics, free of charge and easily accessible from work.* [Physiotherapist FG3-2]
Exemplar 6: Access to keynotes	*If I see a topic I may not be interested in particularly, but I see one of these people have commented, I may then read the original message and a few other comments—this gives me a quick gist of the flow of the topic, I then read the keynote response...I value the high calibre of expertise in the contributors to ICUConnect, thereby I am able to rely on information provided, or at least follow their guidance to view recommended sites to research.* [Clinical nurse—external FG3-1]
Exemplar 7: Overcoming clinical silos	*We all can get caught up in our “own world” and then we never progress, so this world allows QI to progress via discussion and research among like groups in a more timely manner.* [Clinical nurse external FG3-1]

**Table 3:** Belonging to a broader intensive care community: Elements 3 to 5.

Elements 3-5	Exemplar
Exemplar 8: Joint reality	*Innocent questions arise all the time and it is comforting to know that others are thinking along those same lines and asking those same questions. Some of the problems other units have made me realise I am not alone.* [Clinical nurse FG2-2]
Exemplar 9: Water cooler	*There are often interesting topics of discussion and I find that questions I have may have already been answered or ideas posed that I then take to the next level of investigation. Because I work in a small unit, with very limited resources, I find the discussions useful for formulating plans of where we should be heading. The value of this type of information sharing cannot be overstated, particularly for smaller units.* [Equipment NUM KI-1]
Exemplar 10: 24/7 conference	*Joining ICUConnect allows me to do this (gain other perspectives) from those working in the field, without having to take time out from work. I can access limited PD/study leave with virtually no funds available, so this allows me to make a contribution where appropriate on topics I can contribute to, sharing my expertise.* [Physiotherapist FG3-2]

#### Subtheme—Enhanced Access to Best Practice Knowledge

The second subtheme, *Enhanced access to best practice knowledge* (see Figure 1), represents how ICUConnect facilitates innovation access for members and comprised 3 elements: access to credible knowledge, being able to benchmark practice, and keeping up to date. Access to credible knowledge was a minor reason cited by participants when initially asked why they joined the VC; however, its prominence increased over the course of discussions. This element had 4 characteristics: (1) broad ranging knowledge, (2) enhanced access, (3) unpacking of clinical practice, and (4) credible information. For several members a bonus was the opportunity to access the expertise of IC leaders, referred to as *keynotes* (also previously discussed under *Community*). The most prominent characteristic of the *credible knowledge* element was access to a broad range of knowledge, including exposure to reported research that enabled participants to develop local practices and resources. When asked what specific knowledge they had obtained from the VC within the last 3 to 6 months, participants identified a comprehensive list of knowledge that included recent practice knowledge, organizational documents, conference information, equipment, and jurisdictional newsletters. Several participants also reported that they archived discussions for later use. For some members, discussions unpacked clinical practices by introducing nuances of practice that were previously unknown or not considered (see Table 4, Exemplar 11). The second characteristic of *Access to knowledge* was that ICUConnect enhanced access to knowledge because it was a superior knowledge source (relative advantage) compared with other methods, with easy access to experts, information *simply arrived* in their email box, and that they could learn from the experience of others (see Table 4, Exemplar 12). The final characteristic was that participants considered the information credible; this was a function of access to experts or keynotes and that the VC was moderated and sponsored by a health department (see Table 4, Exemplar 13; for further exemplars refer to fifth table in Multimedia Appendix 5).

The second element of *Access to knowledge* was the *ability to compare or benchmark local practice or equipment and then improve practice*. This element was another common motivator to join ICUConnect and continued to arise over the course of focus group discussions. It was clear participants understood that it was important to gain this knowledge, including alternative perspectives, from external knowledge sources to ensure local practices reflected broadly accepted best practice. This extended beyond clinical practices to include equipment, resources, and cultural issues (see Table 5, Exemplar 11). Within this element, members sought to understand whether an innovation was worth implementation by using the experiences of fellow members or vicariously evaluating the observability and relative advantage of an innovation (see Multimedia Appendix 2). The last element of *Access to knowledge* was keeping up to date. When asked why they joined ICUConnect, many participants cited wanting to keep up to date with contemporaneous and topical knowledge (see Table 5, Exemplar 10). This was especially important for participants who did not currently work in an IC unit, as it retained a strong ongoing link to the clinical setting.

**Table 4:** Access to knowledge: Element 1—access to credible knowledge.

Access to credible knowledge element	Exemplar
Exemplar 11: Broad ranging knowledge	*I have used posts—I have also kept some of them...I do recall a lot of discussion on high flow oxygenation—pros & cons etc. I found this particularly interesting as we have seen a reduction in the bipap numbers and in some instances, ventilation, because of this modality.* [NUM FG1-1]
Exemplar 12: Enhanced access	*Those letters or conferences that come via the post for me tend to pile up until I get to them, but on computer, email, forums etc are readily available to me at work in down time, I do tend to get to them before I miss the application final date—or I flag them to come up so I don’t forget them. So those that come in the post are often missed as I don’t carry them all with me to request the day off so I can go to them, but I can request the day off immediately when looking at emails at work.* [Clinical nurse external FG3-1]
Exemplar 13: Information is credible	*As a knowledge bowerbird I value the knowledge that flows across without me having to go search for it! As I have said previously it allows me to keep a finger on the pulse and what's happening. In my current role I am on the LHD Policy and Procedure Committee and I find I call on a lot of information from ICUConnect or the ICU Best Practice Project to rebut some of the out of dated practices that people insist on - it gives me the knowledge that things have changed so I can suggest that what they are proposing is now outdated and that they need to do a literature search.***[**Knowledge broker FG1-2]

**Table 5:** Access to knowledge: Elements 2 to 3.

Elements 2-3	Exemplar
Exemplar 9: Benchmark and improve practice	*It is always helpful (and a relief) to know that what your unit is wanting to implement and change is on par with other practices and it is always paramount to explore why certain options are not adopted.* [Knowledge broker FG3-7]
Exemplar 2: Keeping up to date	*I saw ICUConnect as an active forum where current issues/topics would be discussed; it would be a way to keep abreast of what was going on. I think it was some time before I rustled up the courage to reply or ask for anything!***[**Knowledge broker FG2-11]

## Discussion

The aim of this study was to develop an in-depth understanding of why IC HCPs were members of ICUConnect, a closed VC managed by a government agency, that is, why they join and remain a member of the VC and the purpose this plays in their professional lives and how they use the listserv and the application of knowledge sourced via discussions. The key finding was that by being a member of a broader community, they had access to credible best practice knowledge. In this context, listservs were also perceived as superior to more recent social media technology.

### Listserv Technology Remains a Highly Valued and Viable Social Media Platform

ICUConnect was adopted by these participants as the listserv provided a superior way (relative advantage) of communicating with colleagues, was congruent (compatible) with professional values and beliefs, and was relatively easy to use (complexity) in comparison with other social media. These data align with evidence that HCPs prefer closed professional VCs [46] with perceived high usefulness [46,62,74] and low complexity [41,42,74]. Early research on internet technologies would suggest that a contributing factor might be that the study participants were digital immigrants and therefore perceive newer platforms as more difficult to use [72]; however, generational differences in technology use are now under question [75,76]. In addition, it was noted that closed VCs may be a function of the need for privacy and psychological safety in a professional VC [77], with these VC types also favored by teachers [78] and health care consumers [79]. Usability (how intuitive and easy it is for members to interact within a VC) is also an integral component of ongoing community success [74,80] and was reported as an important difference between nonposters and high posters [80]. Although user needs drive individuals to experiment with social media, the perceived innovation characteristics of that media will influence final adoption decisions [77,81]. The ongoing relevance and viability of listservs can be seen its continued use by MEDLIB (a VC established in 1991) [82], the REDIRIS communities by Spanish HCPs and the health literacy discussion list [83].

### ICUConnect Members Are Motivated Professionals Who Are Oriented to Change

All participants appeared to view VC membership as an integral component of professional practice, as it facilitated maintenance of a contemporaneous knowledge base. Almost two-thirds of this small group of experienced HCPs exhibited cosmopoliteness [9] because they used multiple social media channels, placing them within the early adoption groups. Although early evidence indicated limited professional use of social media by HCPs [46,56,84-88], the findings reflect more recent research where frequent posters demonstrated higher use of social media behaviors [74] and also participated in more boundary spanning activities [39]. Although there are inadequate data in this study to specifically categorize participants, their membership of ICUConnect suggest they may belong to the early adopter side of the innovator curve (see Figure 1) because they chose to communicate outside their immediate professional social network This is supported by how participants vicariously experience innovations via ICUConnect, a key characteristic of the early majority [9]. This suggests these HCPs are oriented to change, similar to a population-based study that reported a significant relationship between being open to new experiences, age, and social media use [89].

Although not all study participants were in formal leadership positions, their strategic participation in ICUConnect and use of other social media reflects an external orientation that enables them to identify innovations to incorporate into their local settings [12,90,91]. Absorption and diffusion of knowledge or innovation within an organization is the role of boundary spanners (eg, nursing unit managers or project officers) [17] and knowledge brokers (eg, nurses in education or advanced practice roles) [92]. This important boundary work contributes to organizational interconnectedness, and intellectual and social capital; reflecting necessary conditions if knowledge is to move across structural, professional, and pragmatic boundaries [19,93]. Knowledge-seeking behavior is a subjective norm shared by individuals who participant in online communities [94] and loiter in information neighborhoods [95]. This participation is likely to be strategic [96] because it is time-intensive, which has previously been identified as a barrier to posting [41,42,45]. The involvement and contributions by these individuals are not self-centered acts, rather they reflect the collegiality and altruism found in business [97] and health [38] VCs where organizational knowledge is viewed as a public good to be shared.

### Intensive Care Professionals Are Members of ICUConnect Because They Belong to a Broader Community and They Have Access to Credible Best Practice Knowledge

The *belonging to a broader community* subtheme embodied the social network of ICUConnect, whereas the subtheme *enhanced access to credible knowledge* represented how the VC afforded members a superior knowledge resource compared with traditional sources. Belonging to a broader community of like-minded HCPs was an integral and highly valued component for all members. The Web-based culture was highly regarded by members because of the quality of discussions, collegiality, and informality. The social network also facilitated access to the whole of the IC community and especially to expertise from key individuals, enabling members to overcome the limitations of local clinical silos. Access to a broad range of colleagues, including experts, is an essential and highly valued aspect of both face-to-face [98] and virtual [99,100] HCP CoPs, a characteristic also common across nonhealth virtual CoPs [101]. This thematic finding adds to the current evidence, which suggests that HCPs belong to VCs to augment their access to best practice knowledge so that they remain clinically current with relevant and quality information, develop workplace resources, and benchmark practice [39,43,53,74,102,103]. Of note, this access was vital and important for all member types, not just posting members who were the main focus and participants in previous research [38]. Given that ICUConnect was in its 11th year (when the study was undertaken), these findings align with current data, which emphasize how important the relationship between a positive Web-based culture and a knowledge-sharing ethos is to the continued success of a VC [104-107].

Belonging was identified early as an integral component for a *sense of VC* [49,105,106,108], which influences how VC members develop trust and participate in Web-based knowledge-sharing activities [103-106]. Similar to Rogers’ homophilly [9], *belongingness* is a contextual experience where individuals feel (1) accepted, valued, and secure within a social group; (2) connected or important to the group; and (3) their professional values align with group norms [109]. A sense of belonging creates the necessary community or relational bonds to encourage members to contribute their knowledge and expertise to the VC [44,101]. As a VC evolves, a critical mass of members see the value of sharing, where both diversity and equality are core characteristics of the online community [79]. The core elements of the overarching theme demonstrate that since it was established, ICUConnect has evolved to become a diverse multidisciplinary team social network that facilitates group affiliation by promoting a collegial professional Web-based experience.

### Study Methods: Strengths and Limitations

Strengths and limitations are noted for the study. Rigor in qualitative research is a contentious space [69,110,111], with preferred terms of *trustworthiness* or *confirmability* reflecting the accuracy and comprehensiveness in how data were collected, analyzed, and reported. To support this, an audit trail was maintained, and a clear description of the research process is provided including a thick description of participants.

Several study limitations are noted. There are 3 design elements limiting transferability to the broader population of HCPs: (1) the qualitative design using focus groups and interviews, (2) the Australian IC setting, and (3) that most participants were nurses. A quantitative design, such as a survey, may have garnered a broader representation; however, as previously noted, prior studies using surveys failed to obtain representative samples. This study instead chose to leverage the advantages of online focus groups with learnings from virtual tertiary education [68] and interviews to facilitate participation by a broad range of members, especially the previously under-researched nonposting majority. Another limitation is that the data collected may have been tilted toward positive experiences because participants were current members. A more balanced dataset may have been created by including past members, who may have different perceptions of ICUConnect; however, this was not considered feasible because past members’ email addresses may have changed. This limitation may have been mitigated by specifically asking about positive and negative experiences.

A key goal of qualitative research is developing rich data and undertaking analysis that leads to findings that reflect participant experience of the phenomenon of interest. The asynchronicity of the focus groups supported moderation, researcher and participant reflexivity, and data quality and analysis. A lack of interaction in the non- and high-posting focus groups was a threat to data quality, although this was partially offset by planned strategies, which increased participation. Despite this planning, the small number of participants in the high-posting focus group [66] did reduce the contributions and interaction of this important cohort. To a limited extent, the key informant interviews may have minimized this limitation. The choice to collect data as discussion threads was a key strength and contributed significantly to study credibility and trustworthiness; namely real-time participant-controlled data collection, ensuring accurate data. Participants were also able to contribute when they wished, as discussions were not taken over by dominant talkers or experts [65], and participants had time to consider their own and previous responses, contributing to rich reflexive responses. Data analysis was enhanced by early immersion [64,70] and more time to record field notes, enabling comparison and contrasting of responses. The moderator was, therefore, able to review and reflect on responses and where appropriate, refer to participants, facilitating both member checking and early theme development.

### Implications

Since the internet was established, all sectors, including business, health care, information technology, and education, have been concerned with designing VCs that optimize the user experience and achieve diverse goals such as information or resource sharing, professional development, or leveraging expertise [107,112]. The critical design elements have been established [113,114]; however, developing a bespoke platform may not ensure acceptance by a target population [115-118]. By using the DOI as the theoretical lens, this study has identified 2 antecedent factors crucial to a successful health care VC, specifically that members of the target population have an external orientation and the chosen platform is compatible with their professional norms. This implies that before implementing a VC, an organization should investigate if the intended target population have a desire to communicate with their professional colleagues using Web-based methods and which platform is acceptable.

### Conclusions

The key study finding was these HCP participants were members of ICUConnect because they had access to a broader IC community, enhancing access to credible, contemporary best practice knowledge. This was a strategic move as participants understood to provide up-to-date clinical practices, they needed access to the knowledge and experience of a broad range of their colleagues. Importantly, it appeared that ICUConnect, as a closed specialty-specific VC, established a broad heterogeneous social (professional) network to overcome the current ineffective networks that adversely impact on knowledge exchange and creation in contemporary local practice settings.

## Appendix

Multimedia Appendix 1 Diffusion of Innovations—innovation adoption curve.

Multimedia Appendix 2 Terms used in Diffusion of Innovation (from protocol).

Multimedia Appendix 3 Recruitment email (from protocol).

Multimedia Appendix 4 Methods.

Multimedia Appendix 5 Participants.

## Abbreviations

CoPs: communities of practice
DOI: Diffusion of Innovation
HCP: health care professional
HREC: Human Research Ethics Committee
IC: intensive care
VC: virtual community
