# Supplementary figures and images for: Why Health Care Professionals Belong to an Intensive Care Virtual Community: Qualitative Study
Source: J Med Internet Res. 2019 Nov 5;21(11):e14068. doi: 10.2196/14068 (PMC6864486; doi:10.2196/14068)

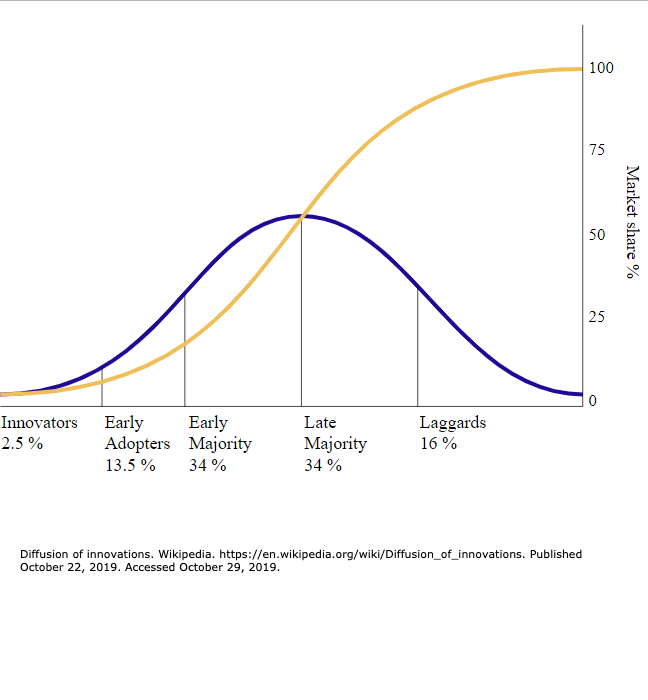

Supplement: Multimedia Appendix 1 [file jmir_v21i11e14068_app1.png]
