# Supplementary material for: Why Health Care Professionals Belong to an Intensive Care Virtual Community: Qualitative Study
Source: J Med Internet Res. 2019 Nov 5;21(11):e14068. doi: 10.2196/14068 (PMC6864486; doi:10.2196/14068)
Supplement: Multimedia Appendix 2 [file jmir_v21i11e14068_app2.pdf]

**Multimedia appendix 1 Description of terms used in Diffusion of innovation framework**

| Aspect of Diffusion of innovation | Characteristic | Descriptor |
|---|---|---|
| Innovation [1] | Relative advantage | The degree to which an innovation is perceived to be an improvement on what it supersedes |
| | Complexity | The degree to which an innovation is perceived to be challenging to comprehend or use |
| | Compatibility | The degree to which the innovation is perceived to be congruent with values, experiences and needs of potential adopters |
| | Trialability | Whether an innovation can be trialled before making an adoption decision |
| | Observability | Whether the outcomes of adoption of an innovation are visible to potential adoptees |
| Individual – Adopter<br><br>Innovators – 2.5%<br>Early adopters – 13.5%<br>Early majority – 34%<br>Late majority – 34%<br>Laggards – 16% | Socioeconomic | Early adopters generally have more years of formal education, a higher social status and higher disposable income [1] |
| | Personality | Early adopters are better able to deal with the abstract, uncertainty and risk, have more empathy and are less dogmatic than later adopters [1] |
| | Personality | Early adopters are better able to deal with the abstract, uncertainty and risk, have more empathy and are less dogmatic than later adopters [1] |
| | Social network | Early adopters have larger and more varied communication channels (including mass media) which extend beyond local boundaries [1] |
| Social system [1] | Definition | Is the patterned arrangement formed by the communication channels between individuals engaged in joint problem solving towards a common goal |
| | Homophilly | The degree to which individuals who communicate are similar (that is share beliefs, values, socioeconomic status and education). A highly homophilous social system aids knowledge diffusion however new knowledge will be difficult to access as redundancy is reached quickly |
| | Heterophilly | The degree to which individuals who communicate are different (that is share beliefs, values, socioeconomic status and education). A highly heterophillic social system will have greater access to novel knowledge but diffusion will be made difficult due to lack of individual trust |
| | Communication | Interpersonal communication channels are where information is |

| Aspect of Diffusion of innovation | Characteristic | Descriptor |
|---|---|---|
| | channels | exchanged between two individuals whereas mass media channels involve information exchanged from one to many. While mass media channels enable access to a broader range of information interpersonal channels, or peer-to-peer knowledge exchange are more influential on decisions to try and then finally adopt an innovation [1] |
| Organisation | Centralisation | degree to which power and control are concentrated in few individuals [1] |
| | Inter-connectedness | Degree to which separate units are in the organisation are linked by social networks [2] |
| | Organisational slack | degree to which uncommitted resources are available [1] |
| | External Orientation | Where organisational leaders have professional networks external to their workplace [3, 4] |
| | Formalisation | degree to which an organisation emphasises rules and regulation [1] |
| | Complexity | degree to which members possess a high level of knowledge and skill [1] |
| | Absorptive capacity | ability of an organisation to acquire, assimilate and exploit new knowledge for organisational advantage[5] |
| | Complexity | degree to which members possess a high level of knowledge and skill [1] |

References


1. Rogers EM. Diffusion of Innovations. 5th ed. New York: Free Press; 2003. ISBN: 978-0-7432-2209-9
2. Nieves J, Osorio J. The role of social networks in knowledge creation. Knowledge Management Research and Practice 2012;**00**:1-16 1477–8238/12
3. Aarons GA, Hurlburt M,Horwitz SM. Advancing a conceptual model of evidence-based practice implementation in public service sectors. Administration and Policy in Mental Health and Mental Health Services Research 2011;**38**(1):4-23.14 December 2010, PMC3025110
4. Purcell R McGrath F. The Search for External Knowledge. Electronic Journal of Knowledge Management 2013;**11**(2):158-167.
5. Ehin C. Hidden assets: harnessing the power of informal networks. London: Kluwer Academic Publishers; 2004. ISBN: 1-4020-8081-6
