# Supplementary material for: Why Health Care Professionals Belong to an Intensive Care Virtual Community: Qualitative Study
Source: J Med Internet Res. 2019 Nov 5;21(11):e14068. doi: 10.2196/14068 (PMC6864486; doi:10.2196/14068)
Supplement: Multimedia Appendix 3 [file jmir_v21i11e14068_app3.pdf]

Multimedia Appendix 2: Online recruitment – demographics and group rules

1. What is your IC-VC email address<br>This will be used to identify how many times you have posted in the last two years (Sept 1 2012-August 312014). This will be used to place you into one of three focus groups<br>• Focus group 1 – posted > 5 times<br>• Focus group 2 – posted ≤ 5 times<br>• Focus group 3 – have not posted	
2. Please select the healthcare professional?	○ Nurse (go to 2)<br>○ Doctor<br>○ Physiotherapist<br>○ Pharmacist<br>○ Dietitian<br>○ Occupational therapist<br>○ Healthcare manager<br>○ other
3. For nurses only – please indicate what best describes your primary professional role	○ Clinical care of patients within a designated unit<br>○ Clinical care of patients across the hospital<br>○ Education of staff within a unit or hospital<br>○ Tertiary education<br>○ A combination of research, practice development or education<br>○ Management of a designated clinical unit/s<br>○ Management across a healthcare facility<br>○ Not a nurse
4. How long have you been a healthcare professional?	
5. Please indicate the best description of your primary workplace	○ Adult ICU/HDU (includes sub specialities)<br>○ Paediatric ICU<br>○ Emergency department<br>○ Coronary care<br>○ Not critical care – please describe
6. How long have you been working in critical care? If not in critical care please move onto question 6	
7. What is your primary place of employment	○ Public hospital<br>○ Private Hospital<br>○ Health department unit<br>○ Healthcare industry<br>○ Tertiary education facility<br>○ Other (please indicate where)
8. What is the location of your primary place of employment	○ NSW<br>○ Victoria<br>○ Queensland<br>○ Western Australia<br>○ South Australia<br>○ Tasmania<br>○ Northern Territory<br>○ Outside Australia (please indicate which country)<br>○
9. Please review the Focus group ground rules and identify whether you agree or disagree.<br>i. I will keep my comments focused on the specific question and use professional language and spelling. However I will use emoticons, capitals or punctuation where I wish to add emphasis to my posts.<br>ii. I will not make personal derogatory comments about the content of other focus group participants' posts<br>iii. Where I discuss the online behaviour of ICUConnect members I will use professional language and not be personally derogatory about any individual<br>iv. I will not discuss the content of any focus group discussions with other colleagues<br>v. I will not disclose the participation of other focus group members to colleagues	
10. Are there any other ground rules you believe are important? Additional rules will be discussed at the beginning of the focus group.	○
