# Supplementary material for: Why Health Care Professionals Belong to an Intensive Care Virtual Community: Qualitative Study
Source: J Med Internet Res. 2019 Nov 5;21(11):e14068. doi: 10.2196/14068 (PMC6864486; doi:10.2196/14068)
Supplement: Multimedia Appendix 4 [file jmir_v21i11e14068_app4.pdf]

Multimedia Appendix 3

Table 1 Apriori moderating plan

Notes from e-moderation [1]			Actions
Stage 1 – Access & motivation (resource 1)	Technical support	Instructions on accessing discussion forum	Provide how to instructions<br>Use of standard subject heading<br>Notification of new question via email
	Motivating participants	Present benefits<br>Try to create fun<br>Address anxiety – provide reassurance	Respond to direct question<br>Provide feedback<br>Provide reassurance
	E-moderating	Welcome participants<br>Log on regular	Welcome participants<br>Log-on twice per day<br>Use of supportive language & emojis
Stage 2 – socialization	Technical support	Explain carefully	Provide sign-posts for how focus group will work
	Learning	Enhance confidence by acknowledging post	Acknowledge responses
	E-moderation	Explain and clarify your role	Explain and clarify your role
Stage 3 – information exchange	Technical	Provide ongoing technical support	As Per stage 1
	E-moderating	Provide relevant information	Ask clarifying questions
Stage 4 – Knowledge construction	Technical support	Ensure good signposting<br>Deal with ongoing technical issues	As Per stage 1
	Learning	Post insightful questions to promote reflection<br>Encourage contributions not just reading	Where applicable provide context around questions
	E-moderation	Seek to establish group understanding	Summarize input

Multimedia Appendix 3

Table 2 Question guide

Question type	Index question guide used for Focus Group 2	Revised question guide used for focus groups 3 and 1, and key informant interviews
Introductory	*1.* Please introduce yourself and tell the group about your professional role and experience	*1.* Please introduce yourself and tell the group about your professional role and experience
Transition	*2.* You were invited to this focus group because you are a member of ICUConnect. Could you explain what prompted you to join?	*2.* You were invited to this focus group because you are a member of ICUConnect. Could you explain what prompted you to join?
Transition	*3.* Do you use any other social media or online communities for professional networking and development?	3. 3 ICUConnect is a listserv, one of the oldest kinds of social media platforms.<br>a. Do you use any other social media or online communities for professional networking and development?<br>b. b. Does ICUConnect have any advantages or disadvantages to these?
Key	4. What do you value most about ICUConnect?	
Key	5. What are the least valuable aspects of ICUConnect	4. What do you value most about ICUConnect? And what do you value least about ICUConnect?
Key	*6.* What advantages or disadvantages does ICUConnect have over other social media?	
Key	7. Current research indicates that there are active users of virtual communities (individuals who post) and passive users (individuals who mainly read &/or share). How would you describe how you use ICUConnect?	5. Current research indicates that there are active users of virtual communities (individuals who post) and passive users (individuals who mainly read &/or share). As an active/passive user how would you describe your use of ICUConnect?
Key	8. Do you share ICUConnect posts with other professional colleagues?	6. Have you been able to use any posts from the last 6 months of discussions?
Key	9. Is there a post in the past 3 months that has been of high relevance to you?	
Key	10. Have you been able to use any posts from the last 6 months of discussions?	7. Is there a post in the past 3 months that has been of high relevance to you?
Concluding	11. Are there any other important aspects of ICUConnect that we have not discussed?	8. Are there any other important aspects of ICUConnect that we have not discussed?
	In the revised question schedule Questions 1 & 2 were asked in the same thread and question 8 was incorporated into Questions 6 and 7. For Focus group 1 the schedule was further revised with questions 6 and 7 grouped together	

Multimedia Appendix 3

Data analysis process

The six-step data analysis process included:

1. Data immersion through active reading of discussion threads and interview transcripts to identify meanings or patterns. This familiarization commenced during data collection in the dual role of researcher-moderator. As indicated earlier, the principal supervisor monitored focus group discussions as a non-participant observer.
2. Data was coded to the casenode (the participant source), the question node, and then inductive coding to a node. A node was a representation or abstraction of what the researcher perceived the participant meant by the data they provided [2].This process ensured that nodes remained linked to their context and source, informing the audit trail and facilitating evaluation based on participant attributes such as posting frequency and professional role. As nodes were identified a descriptor was included to support systematic coding of theory driven nodes [3].
3. Nodes for each question were reviewed and collapsed into candidate themes. A theme is an abstraction that represents a consistent and significant pattern arising from the data as it relates to the research question/s [3, 4]. A research report was developed and discussed at research team meetings until agreement regarding direction of analysis and nodes and themes was reached.
4. Candidate themes were reviewed to highlight coherent patterns supported by consistent data. An initial thematic map was developed using the key aspect of DoI as temporary groupings, and candidate themes were moved into these groups. Matrix coding was then used to compare and contrast themes and to identify if there were any consistent patterns between different types of members (using attributes from the casenode classification sheet). During this process the whole data set was re-read to confirm consistency in the emergent themes; that is considering whether the data did or did not fit the assigned theme [2]. As part of the research diary the memo function was used to describe each theme, to develop the story of

its place within the broader narrative and add links across to other themes where applicable [3].

5. Theme re-working and refinement was completed by identifying the essence of each theme and determining which aspect of the data it captured. This involved development of a detailed analysis of each theme and its associated sub-themes, and how these interacted to provide the overall structure of the overarching theme [4]. A key aspect of this was consideration of how all the elements fitted into broader narrative being told by the participants [4].
6. Findings were synthesized in a final research report.

References

1. Salmon G. E-moderating: The Key to Teaching and Learning Online. 3rd Edition ed. New York: Routledge; 2011. ISBN: 13: 978–0-203–81668–4 (ebk).
2. Bazeley P, Jackson K. Qualitative Data Analysis with NVivo. Second ed. London: SAGE; 2013. ISBN: 978-1-4462-5655-8.
3. Richards L, Morse JM. Read me first for a user's guide to qualitative methods. Thousand Oaks: Sage; 2013.
4. Braun V, Clarke V. Using thematic analysis in psychology. Qualitative research in psychology. 2006;3(2):77-101. doi: 10.1191/1478088706qp063oa.
