# Supplementary material for: Why Health Care Professionals Belong to an Intensive Care Virtual Community: Qualitative Study
Source: J Med Internet Res. 2019 Nov 5;21(11):e14068. doi: 10.2196/14068 (PMC6864486; doi:10.2196/14068)
Supplement: Multimedia Appendix 5 [file jmir_v21i11e14068_app5.pdf]

Table 1 Description of participant demographics

Participant code	No of VC posts	Healthcare professional type	Professional member type	Primary workplace
**Frequent posters (focus group 1 and key-informant interviews)**				
FG1-1	9	Nurse	[a] NUM	Adult ICU
FG1-2	19	Nurse	[b] Knowledge Broker	Local health district
FG1-3	17	Nurse	[b] Knowledge Broker	State-wide public health service
KI-FP-1	23	Physician	[c] Staff specialist	Adult ICU
KI-FP-2	26	Nurse	[a] NUM	Adult ICU
KI-FP-3	10	Nurse	[b] Knowledge Broker	Adult ICU
KI-FP-4	18	Nurse	[b] Knowledge Broker	Adult ICU
**Low posters (focus group 2)**				
FG2-1	4	Nurse	[d] Nurse Academic	University
FG2-2	2	Nurse	[e] Clinical nurse-internal	Adult ICU
FG2-3	1	Nurse	[d] Nurse Academic	University
FG2-4	4	Nurse	[d] Nurse academic	University
FG2-5	3	Nurse	[f] Clinical nurse-external	Hospital specialty service
FG2-6	1	Nurse	[a] NUM	Coronary care
FG2-7	1	Physician	[c] Staff specialist	Adult ICU
FG2-8	1	Nurse	[e] Clinical nurse-internal	Adult ICU
FG2-9	1	Nurse	[e] Clinical nurse-internal	Adult ICU
FG2-10	1	Nurse	[a] NUM	Adult ICU
FG2-11	2	Nurse	[b] Knowledge Broker	Adult ICU
FG2-12	1	Nurse	[b] Knowledge Broker	Adult ICU
FG2-13	4	Nurse	[d] Nurse Academic	Adult ICU
**Non posters (focus group 3)**				
FG3-1	0	Nurse	[f] Clinical nurse-external	Retrieval service
FG3-2	0	Allied Health	Physiotherapist	Adult ICU
FG3-3	0	Nurse	[a] NUM	Adult ICU
FG3-4	0	Healthcare manager	[g] Healthcare manager	Data Analyst
FG3-5	0	Nurse	[d] Nurse Academic	University
FG3-6	0	Nurse	[b] Knowledge Broker	Office
FG3-7	0	Nurse	[b] Knowledge Broker	Adult ICU

[a] Clinical nurse-internal - provides clinical services within a clinical unit
[b] Clinical nurse-external - provides clinical services across multiple clinical units
[c] Knowledge Broker - job role could include advanced practice, education, research or practice development
[d] Clinical unit manager – manages a defined ward or clinical area
[e] Nurse academic– employed by a tertiary education institution
[f] Healthcare manager - employed in a non-clinical or managerial role in health service

Table 2 Professional and intensive care experience of participants

	Frequent posters (n=7) mean (SD; range)	Low Posters (n=13) mean (SD; range)	Non-posters (n=7) mean (SD; range)	all participants (n=27) mean (SD; range)
Intensive care experience	23.00 (2.70; 20-27)	17.46 (6.46; 7-27)	21.14 (7.96; 12-34)	19.85 (6.44; 7-34)
Professional experience	30.29 (4.25; 23-36)	23.77 (7.24; 12-32)	25.14 (8.13; 15-34)	25.81 (7.26; 12-36)

Table 1 Belonging to a broader intensive care community: Element 1 - Online Culture exemplars

Exemplar 1 Positive discussion
'I think the debate come from having so many different clinicians/ managers/ Ds that there are so many different points to so many questions. The discussion often illuminates all these areas. Being from a rural hospital gives you a different perspective than our city cousins' NUM FG2-10
'I also value most of the discussions, and like FG3-2 would like comments to go to the whole list as they're interesting to read. I don't mind if old topics get dredged up-more because I'm curious what the latest is.' Nurse academic FG3-5
Exemplar 2 Negative discussion
That's slightly disappointing with ICUConnect. The majority of peoples tend not to want to engage in a discussion … I think the majority of members are clinical nurses, who may not be so willing to put their heads up Staff specialist KI-1
Sometimes I feel that the conversation gets 'hijacked' by Senior MO's who get into a 'my way's better than your way' contest which actually becomes quite amusing reading at times Clinical nurse – external FG2-5
Exemplar 3 Collegiality
'I most value the knowledge flow and the collegiality of knowledge sharing. Knowledge broker nurse FG1-3
I find the ICUConnect group very willing to share expertise, resource links and comment, so as an engaged group I feel this is a good example of a virtual community (similar to Twitter in that respectXXX - it helps us connect to likeminded individuals and participate in the conversation (or at least observe it) in bites of time that may otherwise not be useful for much' Physiotherapist FG3-2'
Exemplar 4 Constructive atmosphere
'and I think I like principally the respectful way that people-- or that they visibly deal with queries and questions and so on. And I've seen a few kind of attempts to correct direction through the years, and they've all seemed to be received well and I've agreed with them all. So I guess that it's a respectful environment that people feel really free to ask questions, sometimes over and over and over again.' Knowledge broker KI-3

Table 4 Belonging to a broader intensive care community: Element 2 - Community members

Exemplar 5 Whole of intensive care community
'I joined ICUConnect because I have many friends and colleagues in critical care across NSW and the idea of an online discussion group sounded innovative and a good way to talk and share ideas etc with colleagues across the state, as well as ask questions around practice, staffing issues, educational resources, guidelines etc.' Knowledge broker FG1-3
'Accessibility to a huge pool of fellow clinicians,' Knowledge broker FG2-11
Exemplar 6 Access to keynotes
'I think the value is -- uh, it's also the value of-of resource people, knowing regular posters, I suppose, and people that then become resource for areas of expertise.' Equipment NUM KI-2
S*o, what I like about ICU connect is the opportunity for those with specialised knowledge or opinion in a specific area to share that with others and help raise the standard of practice and patient care.' Nurse academic FG2-3'*
Exemplar 7 Overcoming clinical silos
'The very small number of dedicated children's hospitals and PICUs doesn't easily lend itself to groups like this, so we are forced out into the big wide world. Personally, I have always felt this was a good thing as its easy to become a bit insular. Groups like ICUConnect are great for helping avoid that.' Knowledge broker nurse FG1-3

Table 5 Belonging to a broader intensive care community: Elements 3-5

Exemplar 8 Joint reality
'I would have to say its the professional one I use the most, unquestionably. I actually like the feeling of professional connectedness it brings with it - its always good to know you're not alone and we all struggle with the same conundrums.' Knowledge broker nurse FG1-3
'I can say that the issues being discussed are definitely state-wide, if not world-wide... 'Healthcare manager FG3-4
Exemplar 9 Watercooler
'There are often interesting topics of discussion and I find that questions I have may have already been answered or ideas posed that I then take to the next level of investigation. Because I work in a small unit, with very limited resources, I find the discussions useful for formulating plans of where we should be heading. The value of this type of information sharing cannot be overstated, particularly for smaller units.' Equipment NUM KI-1
Exemplar 10 24/7 conference
'The sharing of knowledge has never stopped at the peer reviewed journals, it has been at the bedside, at inservices, at conferences and educational days, in information journals that don't have peer review...I think this is another forum and one that is highly regarded as a place to get immediate help, not have to wait six to 12 months to get an article on research published.' Nurse academicFG2-13

Table 5 Access to knowledge: Element 1 – access to credible knowledge

Exemplar 11 Broad ranging knowledge
My passion is healthcare ethics, so some of the commentary and amazing research that is posted on ICUConnect is great fodder for my brain Nurse Academic FG2-13.
The newsletters from your governing body have been excellent and the document on Failure to Rescue has informed our risk management team here as we developed a new "escalation of care policy and procedure". Because I saw the document in ICUConnect I was able to bring it forward to our steering group on Escalation of Care. NUM FG3-3
Exemplar 12 Enhanced access
'I mean as good as CIAP is, sometimes it great to hear things just in basic terms from people who have already applied particular practices, that have already gained the knowledge, rather than starting from scratch. I think that's one of the fantastic things about ICUConnect is that you don't actually really have to start anything from scratch.' Knowledge broker KI-4
Exemplar 13 information is credible
I probably would not have been keen to join if ICUConnect was not monitored by people knowledgeable about ICU (and who were prepared to step in to intervene when discussions got out of hand). Academic nurse FG2-1
(Moderator)s role and the role she has played comprise a big part of why I value ICUConnect. I think this is very important. If there was not a strong, linking voice, starting discussions; emailing up-to-date literature; threads; talking points from time to time; there is a big risk that the forum could become defunct or less valuable/accessed by others. Physiotherapist FG3-2

Table 6 Access to knowledge: Elements 2-3

Exemplar 9 Benchmark and improve practice
'Generally I put the question out there when I am interested in new equipment or when I want to get more resources – i.e. more staff etc. I always read the responses and although I might not always agree with opinions, I do take them into account when formulating my plans' NUM FG1-1
'It was also a way of better understanding how practice differed in other ICUs and how we might improve the treatment and delivery of care in our own unit' Nurse Academic FG2-1
Exemplar 10 Keeping up to date
'Initially it was just as an extra resource, and the more I used it, the more I actually found I was getting a lot more up-to-date information from talking and chatting to people on ICU connect, then I could ever find in a book or an article or doing a literature search or anything of the sort. So that's what initially brought me to it, and that's what's maintained my interest in it.' Knowledge Broker KI-FP-4
'Therefore, my link to ICU goes back a ways. I keep up to date via reading and regular conference attendance (ICE & the ASM plus others) but I joined ICUConnect as means to facilitate keeping up to date with hot topics and to see what the buzz is at the coalface. To date I haven't been disappointed.' Nurse academic FG3-5
